# Modulation of functional characteristics of resident and thioglycollate-elicited peritoneal murine macrophages by a recombinant banana lectin

**DOI:** 10.1371/journal.pone.0172469

**Published:** 2017-02-24

**Authors:** Emilija Marinkovic, Radmila Djokic, Ivana Lukic, Ana Filipovic, Aleksandra Inic-Kanada, Dejana Kosanovic, Marija Gavrovic-Jankulovic, Marijana Stojanovic

**Affiliations:** 1 Department of Research and Development; Institute of Virology, Vaccines and Sera–TORLAK; Belgrade, Serbia; 2 OCUVAC–Center of Ocular Inflammation and Infection, Laura Bassi Centres of Expertise; Center for Pathophysiology, Infectiology and Immunology; Medical University of Vienna; Vienna, Austria; 3 Department of Biochemistry, Faculty of Chemistry, University of Belgrade; Belgrade, Serbia; "INSERM", FRANCE

## Abstract

We demonstrated that a recombinant banana lectin (rBanLec), which structural characteristics and physiological impacts highly resemble those reported for its natural counterparts, binds murine peritoneal macrophages and specifically modulates their functional characteristics. By using rBanLec in concentrations ranging from 1 μg to 10 μg to stimulate resident (RMs) and thioglycollate-elicited (TGMs) peritoneal macrophages from BALB/c and C57BL/6 mice, we have shown that effects of rBanLec stimulation depend on its concentration but also on the functional status of macrophages and their genetic background. rBanLec, in a positive dose-dependent manner, promotes the proliferation of TGMs from both BALB/c and C57BL/6 mice, while its mitogenic influence on RMs is significantly lower (BALB/c mice) or not detectable (C57BL/6 mice). In all peritoneal macrophages, irrespective of their type and genetic background, rBanLec, in a positive dose dependent manner, enhances the secretion of IL-10. rBanLec stimulation of RMs from both BALB/c and C57BL/6 resulted in a positive dose-dependent promotion of proinflammatory phenotype (enhancement of NO production and IL-12 and TNFα secretion, reduction of arginase activity). Positive dose-dependent skewing toward proinflammatory phenotype was also observed in TGMs from C57BL/6 mice. However, the enhancement of rBanLec stimulation promotes skewing of TGMs from BALB/c mice towards anti-inflammatory profile (reduction of NO production and IL-12 secretion, enhancement of arginase activity and TGFβ and IL-4 secretion). Moreover, we established that rBanLec binds oligosaccharide structures of TLR2 and CD14 and that blocking of signaling via these receptors significantly impairs the production of TNFα and NO in BALB/c macrophages. Since the outcome of rBanLec stimulation depends on rBanLec concentration as well as on the functional characteristics of its target cells and their genetic background, further studies are needed to investigate its effects under physiological and specific pathological conditions.

## Introduction

Macrophages represent large, morphologically and functionally heterogeneous, immune cell population that is often regarded as a bridge between innate and adaptive immunity [1, 2]. As part of innate immunity, they provide a first line of defense against invading pathogens due to their inherent abilities to phagocyte pathogens and mount various anti-microbial mechanisms. Instead, by acting as antigen-presenting cells, they exert an important role in initiation and shaping of the adaptive immune response [3]. The mode of macrophages activation, their previous immunological experience and surrounding milieu highly influence their functional characteristics [4–8]. Moreover, genetic background-dependent qualitative and quantitative differences in the macrophage responses to external stimuli are reported [9].

Depending primarily on their morphological characteristics, peritoneal macrophages, most often used as a model system in macrophages-related functional studies, are divided into two groups assigned as large peritoneal macrophages (LPMs) and small peritoneal macrophages (SPMs). Beside different morphology, a difference in phenotype and functional characteristics of LPMs and SPMs was marked as well [8, 10]. Although the SPMs are generally considered as more responsive to proinflammatory stimulation, it is difficult to associate a specific type of a response exclusively with LPMs or SPMs as the response to (re)stimulation depends also on genetic background and environment.

There are various ways to activate macrophages; one of the most important ways is the stimulation of macrophages via Toll-like receptors (TLRs), which are able to bind specific evolutionary conserved molecules associated with groups of pathogens, known as pathogen-associated molecular patterns [1]. Sensing of bacterial antigens such as lipopeptides or lipopolysaccharides (LPS) via TLR2 or TLR4, respectively, can initiate the production of bactericidal molecules (reactive oxygen and nitrogen species; ROS and RNS, respectively) and proinflammatory cytokines (IL-12, TNF-α, IL-1, IL-6), and enhance antigen-presenting capabilities of macrophages [11].

As macrophages are part of innate and adaptive immune responses, any bioactive substance able to modulate their functional characteristics can exert strong influence on the characteristics of the immune response. Lectins of various origins are shown to be potent modulators of macrophages’ functioning due to their structural and functional characteristics. They exert modulatory influence upon binding to oligosaccharide motives within structures expressed on the surface of macrophages, including TLRs [12]. Various outcomes of lectin–macrophage interaction have been reported: (i) stimulation of nitric oxide (NO) production as well as production of proinflammatory cytokine IL-12, followed by secretion of IL-10 [13, 14], (ii) creation of pro-Th1 milieu characterized by the enhanced secretion of IFN-γ [15,16,17] and, (iii) creation of pro-Th2 milieu characterized by the enhanced secretion of IL-5 [18].

Banana lectin (BanLec), a mannose-specific lectin that belongs to the jackalin superfamily, is among the lectins which are shown to exert immunomodulatory activity [19–25]. There are several naturally occurring and isolated BanLec isoforms. BanLec produced in *E*. *coli* by recombinant technology and assigned as recombinant BanLec (rBanLec), possesses structural and functional characteristics that highly resemble those reported for its natural counterparts. The alignment of rBanLec sequence to the one of BanLec isolated from natural sources shows a high degree of similarity (~95%) with particular emphasize on the preservation of sequences coding rBanLec’s binding loops [19]. Both BanLec and rBanLec are reported to be a potent mitogen of human and mouse T cells [19, 21, 22] and a promoter of proinflammatory cytokines expression within splenocytes [21, 22]. It is also reported that BanLec initiated enhancement of NO production by macrophages [23], but its influence on macrophages is not extensively evaluated.

In this paper we first evaluated the influence of rBanLec stimulation on the functional characteristics of murine peritoneal resident (RMs) and thioglycollate-elicited macrophages (TGMs) in two genetically different inbred mouse strains BALB/c and C57BL/6; second, we identified rBanLec target membrane structures, specifically TLR2 and CD14; and third, the functional effects of these interactions were examined in fine detail.

## Materials and methods

### Ethics statement

All experiments were performed according to a protocol approved by the EthicsCommittee for the Welfare of Experimental Animals of Institute of Virology, Vaccines and Sera–Torlak (approval no. 20/2016). All experiments conformed to the Serbian laws and European regulations on animal welfare. Every effort was made to minimize animal suffering. Prior collection of peritoneal macrophages, mice were euthanized by cervical dislocation. We did not observe any unexpected deaths of animals during this study.

### rBanLec

rBanLec used in this study is 6His-tagged protein [19]. It was produced in *E*. *coli* SG13009 [pREP4] transformed with expression vector pQE70 (Qiagen, Hilden, Germany), which contained BanLec-encoding insert (GenBank accession number EU055641). The protocol for isolation of total RNA from banana pulp (*Musa acuminata*, Cavendish) and amplification of the BanLec gene is given in Gavrovic-Jankulovic et al., 2008 [19]. Production of rBanLec was performed according to already described procedures [24]. All rBanLec preparations were determined by limulus amoebocyte lysate (Charles River Laboratories, USA) assay to contain an endotoxin contamination of less than 0.5 ng/ml.

### Animals

The research was conducted on BALB/c mice (10–12 weeks old females). All mice were purchased from Institute of medical research at the Military medical academy, Belgrade, Serbia. Animals were housed at the Animal Facility of the Institute of Virology, Vaccines and Sera “Torlak”, kept at a temperature of 21°C under a 12 h:12 h light/dark cycle with *ad libitum* access to water and food. Health status (food and water intake, body weight measurement) of animals was monitored daily by trained animal care staff.

C57BL/6 mice (10–12 weeks old females) were also used but only for specific experiments in this study. As it is already known that L-arginine metabolism and profile of secreted cytokines are highly dependent on the genetic background [9] we decided to evaluate the characteristics of L-arginine metabolism (NO production by nitric oxide synthase (NOS) and urea production by arginase) and cytokine production upon specific BanLec stimulation in BALB/c (Th2-prone) and age-matched C57BL/6 (Th1-prone) mice.

### Macrophages collection

Macrophages were obtained from the peritoneal cavity of mice. RMs, used as a model system for macrophages under resting conditions, were isolated by washing the peritoneal cavity with 5 ml of sterile ice-cold phosphate buffered saline (PBS), pH 7.4. Individual cell suspensions were washed 2 times with RPMI 1640 medium (Sigma, St. Louis, MO, USA) supplemented with 5% fetal calf serum (FCS; PAA, Pasching, Austria) (centrifugation at 300 x g, 10 min at 4°C). TGMs, also known as inflammatory macrophages, were obtained in the same manner from mice that were intraperitoneally injected with 1 ml of sterile 3% thioglycollate medium (Torlak, Belgrade, Serbia) 4 days earlier. The viability of these cell preparations, as determined by trypan blue exclusion, was greater than 95%.

### Cell culture

Aliquots of RMs and TGMs were diluted to the final concentration of 1x10^6^ cells/ml in RPMI 1640 medium and plated to 24-well flat-bottomed tissue culture plates (1 ml of each suspension per well, 1x10^6^ cells/well). Then, the cell suspensions are incubated at 37°C, 5% CO_2_ for 2h, allowing the cells to adhere. Non-adherent cells were discarded by washing the plates twice with warm RPMI 1640 medium.

RMs and TGMs were cultured in RPMI 1640 medium supplemented with 10% FCS, 2 mM L-glutamine, 50 μM β-mercaptoethanol, 100 μg/ml penicillin and 100 U/ml streptomycin, in the presence of different concentrations of rBanLec (1, 5 or 10 μg/ml) or without any stimulation (incubation at 37°C, 5% CO_2_). After 48h incubation, culture supernatants of stimulated and non-stimulated RMs and TGMs were collected and frozen at -70°C until assayed for cytokines.

For NO, arginase and myeloperoxidase (MPO) assays, the cells were seeded (1x10^6^ cells/ml, 100 μl per well) in a flat bottom 96-well tissue culture plates, left the cells for 2h to adhere and cultured in the absence or in the presence of rBanLec (1, 5 and 10 μg/ml). The culture supernatants were collected after 48h of incubation and immediately analyzed for NO. Subsequently, 96-well plates were rinsed twice with warm PBS and centrifuged for 5 min at 250 x g for each wash/rinse step. Cells were lysed with 0.1% Triton X-100 / PBS containing protease inhibitors (50 μl/well) and plates were shaken gently for 30 min at room temperature (RT; 23±1°C). The 96-well culture plates with the obtained cell lysates were frozen at -20^°^C until assayed for arginase and MPO activity.

Additional 96-well tissue culture plates were seeded with RMs and TGMs (100 μl/well, 1 × 10^6^ cell/ml), stimulated and incubated in the same way as above mentioned and used individually for proliferation and nitro blue tetrazolium (NBT) reduction assays.

### Proliferation assay

Proliferative response of RMs and TGMs to rBanLec stimulation was assessed using Cell Counting Kit-8 reagent (CCK-8; Sigma). CCK-8 (10 μl/well) was added into cell cultures upon 48h long incubation, and the cells were incubated for additional 4h. Reactions were stopped by the addition of 1% (w/v) sodium dodecyl sulfate (100 μl/ well), and absorbance values were measured at wavelength of 450/650 nm (A_450/650_) using a spectrophotometer (Multiskan Ascent, Labsystems, Helsinki, Finland).

The number of viable macrophages per well was calculated using a standard curve A_450/650_ = *f* (number of cells). Discrete pool of non-stimulated macrophages was used as standard after counting in the presence of trypan blue (Countess Automated Cell Counter, Invitrogen, Waltham, Massachusetts, USA). Standard suspension was plated in serial dilutions prior to addition of CCK-8 and further treated identically as the experimental wells.

A proliferation index (PI) for each specifically stimulated sample was calculated as the ratio of number of macrophages per well present in stimulated (S) to number of corresponding (RMs or TGMs) viable macrophages per well incubated without stimulation (So), such that PI = S/So.

### MPO assay

Enzymatic activity of MPO was measured in cell lysates of rBanLec-stimulated and non-stimulated RMs and TGMs (section *Cell culture*). This assay is based on the oxidation of *o*-phenylenediamine (Sigma) by MPO. Once the 96-well tissue culture plates with cell lysates were thawed at 37°C, 50 μl of substrate solution (1 mg/ml *o-*phenylenediamine, 0.01% hydrogen peroxide, 50 mM citric acid, pH 5) was added into the wells. The reaction was stopped by the addition of 1 M sulfuric acid and the absorbance was measured at wavelength 492/620 nm (A_492/620_) using an ELISA reader (Multiskan Ascent).

### NBT reduction assay

Intracellular generation of superoxide anion (O_2_^-^) was measured by NBT reduction assay [26]. after 48h long incubation RMs and TGMs with rBanLec in discrete concentrations or without any stimulation.

### NO assay

Activity of NOS was measured via the production of NO. Nitrite, a stable NO metabolite, was quantified in the culture supernatants by Griess reagent [27].

### Arginase activity

Arginase activity was determined for cell lysates of rBanLec-stimulated and non-stimulated RMs and TGMs (section *Cell culture*) by measuring the concentration of urea, a conversion product of L-arginine, as previously described [28].

### Cytokine assay

The production of IL-4, IL-10, IL-12, TNFα and TGFβ in culture supernatants was determined by sandwich ELISA, using commercially available monoclonal Abs, as it is described in Marinkovic et al. [24].

### Identification of rBanLec-targeted structures by Western blot

The mouse peritoneal TGMs (5x10^6^ cells/ml) were washed twice with ice-cold PBS, pelleted, and lysed for 30 min, 4°C in 300 μl of buffer containing 150 mM NaCl, 10 mM Tris-HCl (pH 7.5), 2 mM Na_2_EDTA, 1% Triton X-100, 0.5% NP-40 (RIPA lysis buffer; ThermoFisher Scientific, Waltham, Massachusetts, USA) and protease inhibitors. After centrifugation at 14.000 x g (15 min at 4°C), supernatants containing whole cell extract were mixed with SDS-sample buffer (62.5 mM Tris-HCl, pH 6.8, 2% SDS, 20% glycerol, and 0.04% bromophenol blue). 30 μl of each sample was loaded onto 9% sodium dodecyl sulfate polyacrylamide gel (SDS-PAG) and the electrophoresis (SDS-PAGE) performed with a MiniProtean electrophoresis system (Bio-Rad, Hercules, CA, USA) at 120 V for 2h. After SDS-PAGE, proteins were transferred to polyvinylidene fluoride (PVDF) membrane (Serva, Heidelberg, Germany). Then, the membrane was saturated in 3% skimmed milk/PBS 18 h at 4°C. After washing with PBS containing 0.05% Tween 20, the membrane was incubated with 10 μg/ml of biotinylated rBanLec prepared in 3% skimmed milk/PBS for 1h. The proteins were detected using extrAvidine-alkaline phosphatase/5-Bromo-4-chloro-3-indolyl phosphate/NBT system.

Western blot analysis was also performed to rule out the presence of TLR2, TLR4 and CD14 in lysates used in ELISA. TLR2-, TLR4- and CD14-specific monoclonal antibodies were used in these experiments (Biolegend, San Diego, CA, USA).

### Cell immunostaining and confocal microscopy

Isolated peritoneal RMs and TGMs were centrifuged at 300 x g, 10 min at 4°C and resuspended in Ca^2+^/Mg^2+^-free Hank balanced salt solution to the final concentration of 1x10^5^ cells/ml. For each cell suspension was prepared a cytospin slide (centrifugation 250 x g, 10 min, onto glass slides coated with poly-L-lysine, dried for 1h at RT, fixed in cold acetone for 10 min, and stored at -20°C). For staining, slides were rehydrated for 5 min in PBS and then blocked with 3% skimmed milk in PBS for 30 min. Then, slides were incubated individually with 3 μg/ml of anti-TLR2-FITC Ab (Biolegend) or anti-TLR4-FITC Ab (Biolegend), anti-CD14-FITC Ab (Biolegend) and the appropriate isotype controls (diluted in 3% skimmed milk in PBS), 18 h at 4°C. After three washes in PBS, biotin-labeled rBanLec was added to each slide (3 μg/ml dissolved in 3% skimmed milk/PBS). The slides were incubated 2h at RT and after tree washes with PBS, the slides were stained with streptavidine-rhodamineB (diluted 1:60 in 3% skimmed milk/PBS; Sigma) and incubated for 1h at RT. After rinsing in PBS, the slides were mounted with Fluorescent Mounting Medium (Dako, Glostrup, Denmark). Images were captured by inverted confocal microscope Zeiss Laser scanning system LSM 510 Meta (Carl Zeiss, Jena, Germany) and processed with Zeiss LSM Image Browser software.

### ELISA-based testing of rBanLec binding to TLR2, TLR4 and CD14

Lysates of TGMs were prepared using RIPA lysis buffer (ThermoFisher Scientific) with protease inhibitors and, were added to the 96-well microtiter plates (MaxiSorp, Nunc) previously coated with 10 μg/ml anti-TLR2 Ab, anti-TLR4 Ab and anti-CD14 Ab, separately (50 μl/ml, 18 h at 4°C). Following four washes with PBS after each incubation step, biotin-labeled rBanLec (10 μg/ml), previously incubated with and without 0.5 M methyl-α-D-mannopyranoside was then added to the wells. Individual interactions of rBanLec with TLR2, TLR4 and CD14 were visualized with extrAvidin-alkaline phosphatase/p-nitrophenyl phosphate system (Sigma). The absorbance was measured at wavelength of 405 nm (A_405_).

### Macrophages culturing in the presence of TLR2- and CD14-specific blocking antibodies (neutralizing test)

RMs and TGMs (1x10^6^ cells/well, 24-well flat-bottomed tissue culture plates) were pre-incubated (1h at 37°C) separately with 20 °g/ml anti-TLR2 (clone T2.5, Biolegend) and 20 °g/ml anti-CD14 (clone M14-23, Biolegend) monoclonal Abs before stimulation with different concentrations of rBanLec (0, 1, 5 and 10 °g/ml). Used monoclonal Abs have been declared to block signaling via TLR2 and CD14, respectively. After 48h incubation at 37°C, 5% CO_2_, culture supernatants of rBanLec-stimulated and non-stimulated RMs and TGMs were collected and frozen at -70°C until assayed for cytokines (procedure as described in section *Cytokine assay*). Aliquots of each supernatant (100 °l) were immediately tested for NO (procedure as described in section *NO assay*).

RMs and TGMs stimulated individually with peptidoglycan (PEPG; 10 °g/ml) and LPS (100 ng/ml; agonists for TLR2 and TLR4/CD14, respectively) were used as a positive control of macrophages activation.

### Statistical analysis

The results are presented as mean value ± standard error (SE) calculated for at least three independent measurements. The statistical significance of the observed differences was evaluated using one-way repeated ANOVA followed by Bonferroni’s multiple comparison test.

## Results

### rBanLec acts as a mitogen for RMs and TGMs

rBanLec, in concentrations ranging from 1 to 10 μg/ml, induced proliferation of RMs and TGMs from BALB/c mice. Viable cells counts for both types of macrophages upon rBanLec stimulation were significantly increased in comparison with the corresponding non-stimulated culture (*P<*0.05 for RMs and *P<*0.005 for TGMs).

**Fig 1:**
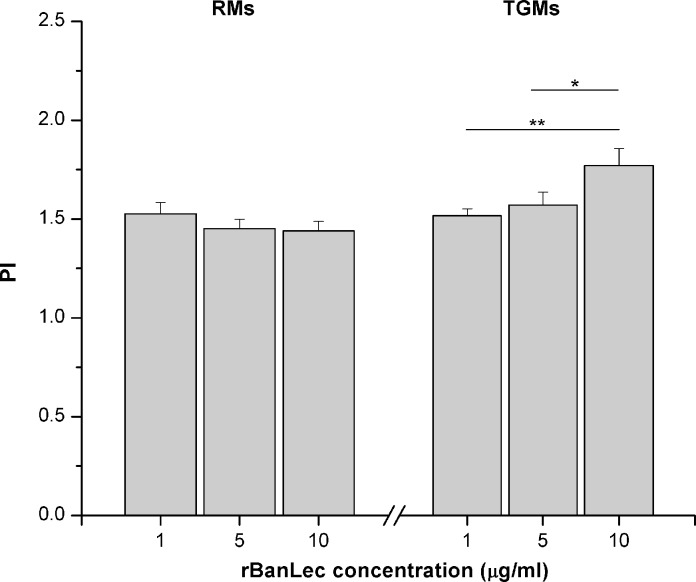
Proliferation index (PI) of rBanLec-stimulated peritoneal RMs and TGMs from BALB/c mice. Macrophages were incubated without any stimulation or with 1, 5 and 10 μg/ml rBanLec. Number of viable cells was determined by CCK-8 assay after 48h long incubation and used for PI calculations. The results were presented as mean PI ± SE. The significance of the observed differences was calculated by one-way repeated ANOVA followed by Bonferroni’s multiple comparison test (*P* <0.05*, *P* <0.005**, *P* <0.0001***). Solid lines indicate compared groups.

Within tested range of concentrations, rBanLec did not significantly influence the proliferation intensity of RMs. On the contrary, TGMs showed a positive, dose-dependent, mitogenic-like response to rBanLec stimulation (*P<*0.005 for 10 μg/ml vs. 1 μg/ml, *P<*0.05 for 10 μg/ml vs. 5 μg/ml; Fig 1). Positive dose-dependent mitogenic-like influence of rBanLec on TGMs was confirmed in C57BL/6 mice (S1 Fig)

### rBanLec alters MPO activity and intensity of superoxide anions generation in macrophages

**Fig 2:**
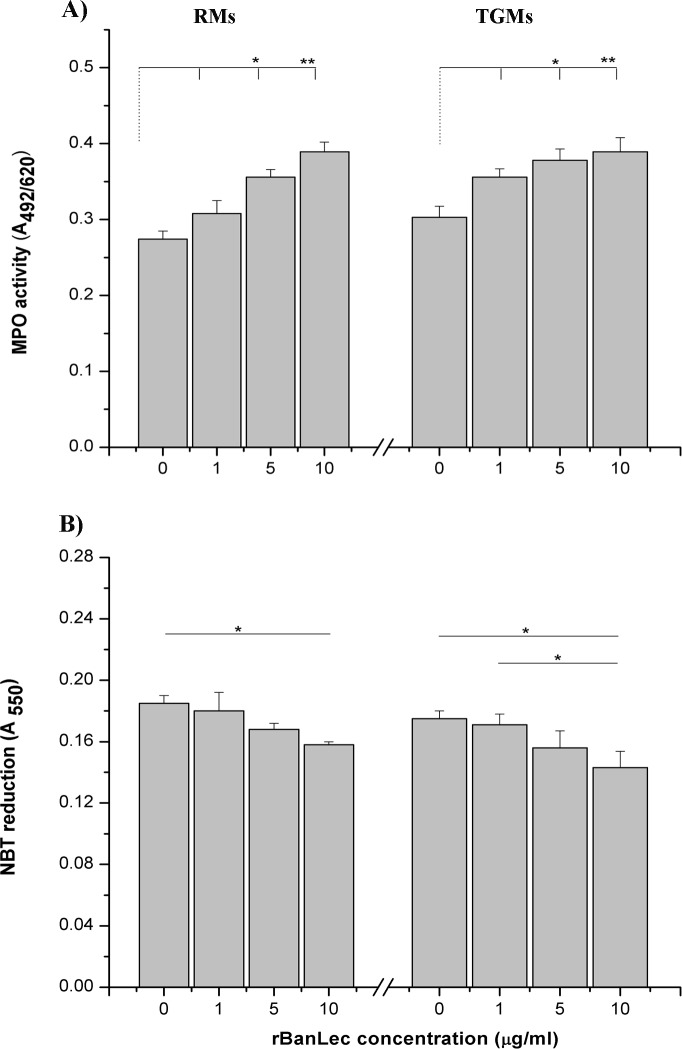
**Activity of MPO (A) and reduction of NBT (B) in peritoneal RMs and TGMs from BALB/c mice stimulated with 1, 5 and 10** μ**g/ml rBanLec or incubated without any stimulation for 48h.** The bars represent mean values of absorbance (A_492/620_ or A_550_) ± SE. Statistical significance of the observed differences was evaluated using one-way repeated ANOVA followed by Bonferroni’s multiple comparison test (p <0.05*, p <0.005**, p <0.0001***). Solid lines indicate compared groups.

rBanLec in concentrations ranging from 1 to 10 μg/ml in RMs and TGMs cultures influenced the MPO activity and intensity of O_2_^-^ production (Fig 2). Positive correlation between rBanLec stimulatory concentration and MPO activity was marked in both RMs and TGMs cultures (Fig 2A). Results of NBT reduction assay showed that macrophages, irrespective to their activation status, produced less amount of O_2_^-^ upon rBanLec stimulation. The highest levels of O_2_^-^ were recorded in non-stimulated RMs and TGMs cultures and in rBanLec-stimulated cultures its level negatively correlated to the applied rBanLec concentration (Fig 2B).

### rBanLec influence on arginase activity and NO production depends on the activation status of macrophages

**Fig 3:**
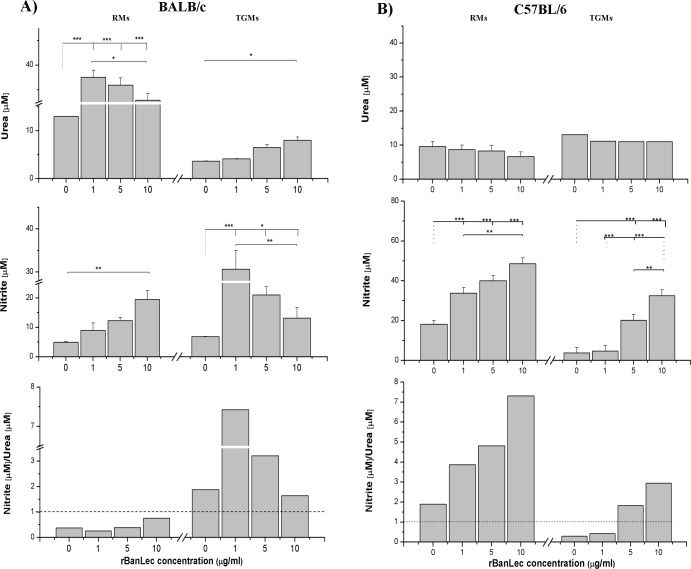
**Activity of arginase and NO production in peritoneal RMs and TGMs from (A) BALB/c and (B) C57BL/6 stimulated with 1, 5 and 10 μg/ml rBanLec or incubated without any stimulation for 48h.** Arginase activity being assessed via measuring the production of urea while NO production was measured using Griess reagents (proportional to the concentration of nitrates). Relationship between NOS (producer of NO) and arginase activities is presented as a ratio of concentrations of their products. Data are presented as mean concentration ± SE. The significance of the observed differences was calculated by one-way repeated ANOVA followed by Bonferroni’s multiple comparison test (*P* <0.05*, *P* <0.005**, *P* <0.0001***). Solid lines indicate compared groups.

The influence of rBanLec on NO production and arginase activity were assessed on RMs and TGMs obtained from two genetically different mouse strains: BALB/c (Fig 3A) and C57BL/6 (Fig 3B).

rBanLec-stimulation significantly elevated arginase activity in all RMs from BALB/c mice cultures (*P<*0.001 vs. non-stimulated RMs) and it was negatively correlated to the applied rBanLec concentration. On the contrary, a positive dose-dependent enhancement of NO production was marked with rBanLec-stimulated RMs from BALB/c mice (*P<*0.05 for 10 μg/ml vs. non-stimulated RMs). In TGMs from BALB/c mice, basal NO production was higher, while arginase activity was lower in comparison to non-stimulated RMs (NO: *P*<0.05, arginase: *P*<0.005). Upon rBanLec stimulation, the highest arginase activity was recorded in TGMs stimulated with 10 μg/ml rBanLec (*P<*0.05 vs. non-stimulated TGMs), while NO production in the same culture was significantly lower comparing to the culture stimulated with 1 μg/ml and 5 μg/ml rBanLec (*P<*0.005 10 μg/ml vs. both 1 μg/ml and 5 μg/ml).

rBanLec, in the same concentration range, significantly enhanced NO production in both RMs and TGMs from C57BL/6 mice; the enhancement positively correlated with the applied rBanLec concentration. Contrary to the BALB/c macrophages, the enhancement of arginase activity upon rBanLec stimulation was not detected in both RMs and TGMs from C57BL/6 mice (Fig 3B). In addition to the analysis of dose-dependent influence of rBanLec on NOS and arginase activities, we also investigated the ratios of their activities within a particular culture in order to determine which enzyme activity is a dominant one. The obtained results showed that in all cultures of RMs from BALB/c mice the arginase dominated over the NOS activity while the opposite situations were recorded for RMs from C57BL/6 mice. In TGMs, NOS activity dominated over the activity of arginase in all cultures of TGMs from BALB/c mice and in TGMs from C57BL/6 mice stimulated with 5 μg/ml and 10 μg/ml rBanLec.

### rBanLec stimulation differently influences the cytokine secretion pattern in RMs and TGMs

**Fig 4:**
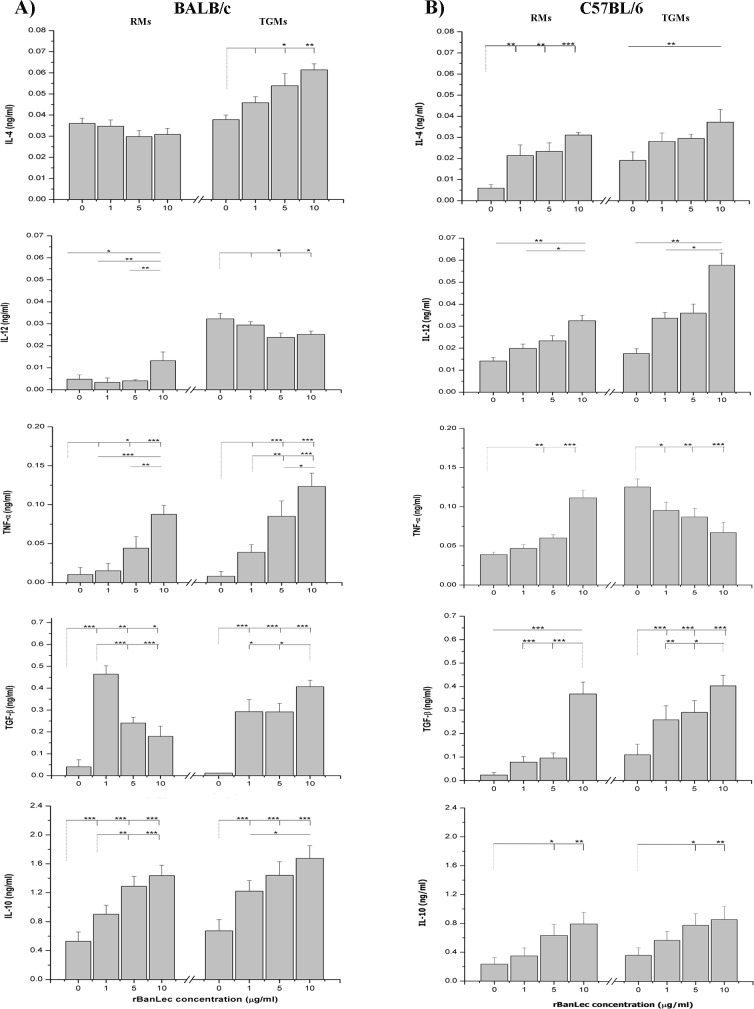
**Production of IL-4, IL-12, TNF-α, TGFβ and IL-10 by RMs and TGMs from (A) BALB/c and (B) C57BL/6 stimulated with 1, 5 and 10** μ**g/ml rBanLec or incubated without any stimulation for 48h.** Concentration of cytokines is determined in supernatants by ELISA. All data are presented as mean concentration of cytokine ± SE. The significance of the observed differences was calculated by one-way repeated ANOVA followed by Bonferroni’s multiple comparison test (p <0.05*, p <0.005**, p <0.0001***). Solid lines indicate compared groups.

To determine whether the treatment with different concentrations of rBanLec would promote RMs and TGMs from BALB/c and C57 BL/6 mice to produce cytokines, the supernatants of these cultures were analyzed (Fig 4). The constitutive production of IL-4, TNFα, TGFβ, and IL-10 was comparable in RMs and TGMs from BALB/c mice, while constitutive production of IL-12 by TGMs was significantly higher in comparison to RMs (*P*<0.001; Fig 4A). As depicted at Fig 4A, rBanLec stimulation exerted the opposite effects on the production of IL-4, IL-12 and TGFβ in BALB/c RMs and TGMs. The production of effector cytokines (IL-4 and IL-12) in RMs from BALB/c mice were affected only upon stimulation with 10 μg/ml rBanLec–comparing to the non-stimulated RMs IL-4 secretion was diminished, while IL-12 secretion was enhanced (*P*<0.05; Fig 4A). In all tested concentrations, rBanLec modulated the production of IL-4 and IL-12 in TGMs from BALB/c mice–a dose-dependent enhancement was marked in the case of IL-4 (*P<*0.05 for 5 μg/ml rBanLec vs. non-stimulated TGMs and *P<*0.005 for 10 μg/ml rBanLec vs. non-stimulated TGMs; Fig 4A), while constitutively high IL-12 production was reduced in a dose-dependent manner within rBanLec-stimulated cultures (*P<*0.05 for 5 μg/ml and 10 μg/ml rBanLec vs. non-stimulated TGMs; Fig 4A). Production of TGFβ was enhanced in all BALB/c-originated rBanLec-stimulated cultures comparing to the non-stimulated corresponding cultures (*P<*0.05). However, the comparison of TGFβ concentrations recorded upon rBanLec stimulation of RMs from BALB/c mice showed negative correlation to the applied rBanLec concentration, while a positive correlation was marked in rBanLec-stimulated TGMs (Fig 4A). Further, rBanLec stimulation, in a positive dose-dependent manner, enhanced the production of IL-10 (*P<*0.01 for all rBanLec-stimulated vs. corresponding non-stimulated; Fig 4A) and TNFα (*P<*0.05 for 5 μg/ml rBanLec and 10 μg/ml rBanLec vs. corresponding non-stimulated; Fig 4A) in both RMs and TGMs from BALB/c mice. In BALB/c mice, the stimulatory influence on TNFα production was more pronounced in TGMs in comparison with RMs.

RMs and TGMs from C57BL/6 mice secreted the same levels of IL-10 and IL-12, while production of other tested cytokines was higher in TGMs cultures (Fig 4B). Besides, in both rBanLec-stimulated RMs and TGMs from C57BL/6 mice, a positive dose-dependent enhancement in secretion of cytokines was marked for all tested cytokines except in TNFα cultures (Fig 4B). Comparing to the corresponding non-stimulated cultures, the enhanced secretion of TNFα was recorded in RMs stimulated with 10 μg/ml rBanLec (*P*<0.001; Fig 4B), while the production of TNFα was diminished in all rBanLec-stimulated TGMs cultures from C57BL/6 mice (*P*<0.005; Fig 4B).

### rBanLec binds the oligosaccharide structures of TLR2 and CD14

**Fig 5:**
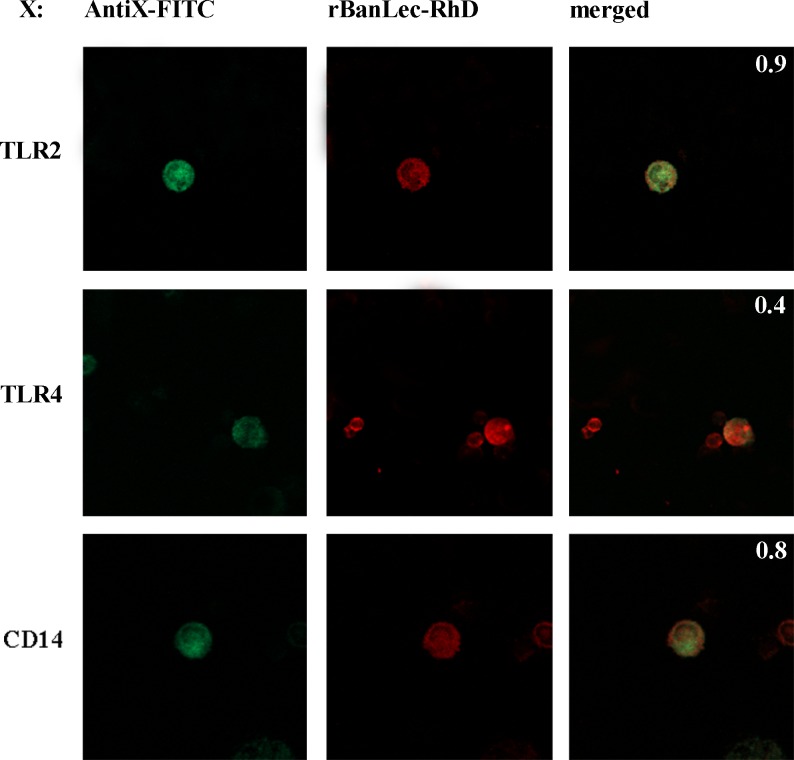
Co-localization of rBanLec binding with TLR2, TLR4 and CD14 expressed on peritoneal macrophages. TLR2, TLR4 and CD14 were detected with FITC-labeled monoclonal antibodies (green color): anti-TLR2-FITC antibody, anti-TLR4-FITC antibody and anti-CD14-FITC, respectively. rBanLec (biotin-labeled) was detected with streptavidine-rhodamineB (red color). Staining of the cells with adequate isotype control antibodies as well with streptavidin-rhodamine, rBanLec- streptavidin-rhodamineB and FITC-labeled antibodies are presented in S2 Fig. The yellow color in the merged image and overlap coefficient (upper right corner of merged image) indicate the extent of the co-localization of rBanLec binding with TLR2, TLR4 and CD14.

To identify structure(s) on macrophages targeted by rBanLec we combined Western blot, ELISA-based testing and confocal microscopy. Preliminary experiment, where lysates of TGMs were resolved by reducing SDS-PAGE and assessed by Western blot for recognition by rBanLec, showed multiple-lane reactivity of rBanLec (S3 Fig). Confocal microscopy showed that rBanLec intensively co-localizes with TLR2 and CD14 but not with TLR4 on the surface of both RMs and TGMs (a representative staining is depicted in Fig 5).

**Fig 6:**
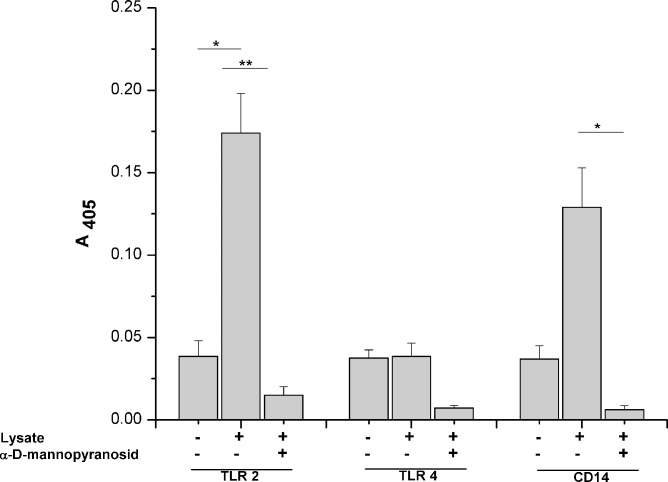
Binding of rBanLec to TLR2, TLR4 and CD14 –the effect of methyl-α-D-mannopyranoside. TLR2, TLR4 and CD14 were extracted from TGMs lysate with specific monoclonal antibodies adsorbed onto microplate. Biotin-labeled rBanLec (10 μg/ml), pre-incubated with or without 0.5 M methyl-α-D-mannopyranoside (α-D-Man), was added to the wells. Alkaline phosphatase / *p-*nitrophenylphosphate system was used for the visualization of rBanLec binding. Results are presented as mean A_405_ ± SE. The significance of the observed differences was calculated by one-way repeated ANOVA followed by Bonferroni’s multiple comparison test (p <0.05*, p <0.005**, p <0.0001***). Solid lines indicate compared groups.

To be able to confirm rBanLec binding to TLR2 and CD14 and to explore in fine detail the nature of these interactions, we performed ELISA where Abs specific for mouse TLR2 and CD14 were used as capture Abs and cell lysates prepared from TGMs as a source of TLR2 and CD14 (S4 Fig). We showed that rBanLec bound to TLR2 and CD14 and those interactions were inhibited by *α*-D-mannopyranosid (Fig 6). In line with the results of confocal microscopy, we did not mark any specific rBanLec binding to TLR4 in our ELISA system (Fig 6).

### TLR2 and CD14 are involved in the production of cytokines and NO by rBanLec in macrophages

**Fig 7:**
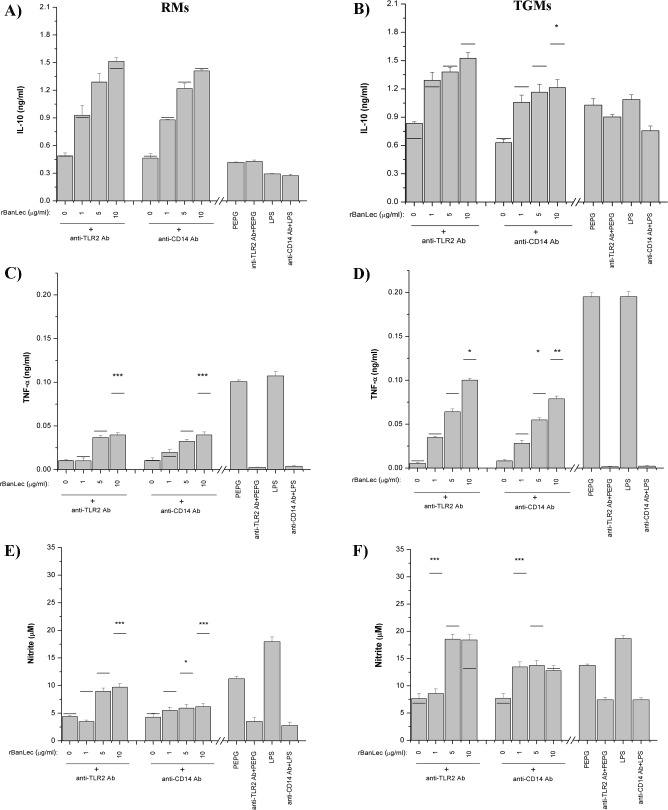
**Impact of TLR2- and CD14-triggered mechanisms in production of IL-10 (A, B), TNFα (C, D) and NO (E, F) upon rBanLec stimulation of peritoneal RMs (A, C, E) and TGMs (B, D, F) from BALB/c.** RMs and TGMs were stimulated with rBanLec (1, 5 and 10 μg/ml) in the presence of anti-TLR2 or anti-CD14 blocking monoclonal antibodies (20 μg/ml) for 48h. Cytokines and NO were measured in supernatant by ELISA and colorimetric method using Griess reagent, respectively. Bars presented mean concentration ± SE. Corresponding mean concentrations of IL-10, TNFα and NO measured upon incubation under the same conditions but without blocking antibodies are indicated by black solid line and are considered as referent. The significance of the observed differences, due to incubation with particular blocking antibody, was calculated by one-way repeated ANOVA followed by Bonferroni’s multiple comparison test (p <0.05*, p <0.005**, p <0.0001***). LPS–lipopolysaccharide, PEPG–peptidoglycan.

To determine whether the NO and cytokines (TNF-α and IL-10) production were mediated through rBanLec-activated TLR2 and (TLR4/)CD14 signaling pathways, we used blocking Abs to these receptors. Stimulation of RMs and TGMs with LPS or PEPG resulted in a dramatic increase of TNF-α and NO concentrations and the increases in TNF-α and NO production were inhibited when TLR2 and CD14 were blocked (Fig 7A, 7B, 7E and 7F, respectively). Inhibition of TLR2 on RMs with blocking Ab (Fig 7C) resulted in up to 55% reduction in TNF-α secretion depending on rBanLec concentrations (10 μg/ml rBanLec, with vs. without blocking TLR2: *P<*0.001). Preincubation of RMs with anti-CD14 Abs (Fig 7C) also affected the production of TNF-α (10 μg/ml rBanLec, with vs. without blocking CD14: *P<*0.001) in the same way as anti-TLR2 Abs. Reduced TNF-α production (ranging from 48–82%) was also confirmed in TGMs when blocking Abs to TLR2 (10 μg/ml rBanLec, with vs. without blocking TLR2: *P<*0.05) and CD14 (with vs. without blocking CD14: *P<*0.05 for 5 μg/ml rBanLec, *P<*0.005 for 10 μg/ml rBanLec) were applied separately, prior to stimulation with rBanLec (Fig 7D). Addition of the same blocking Abs did not affect rBanLec-stimulated IL-10 production in RMs cultures (Fig 7A), while a small reduction of IL-10 secretion was observed in the culture of rBanLec-stimulated TGMs blocked with anti-CD14 antibodies (Fig 7B; 10 μg/ml rBanLec, without blocking CD14: *P<*0.05).

Blocking of TLR2 or CD14 on RMs (Fig 7E) and TGMs (Fig 7F) significantly reduced the production of NO comparing to the corresponding rBanLec stimulated cultures without blocking Abs. The most prominent reduction in NO secretion (~70%) was observed in RMs stimulated with 10 μg/ml of rBanLec (Fig 7E; with vs. without blocking TLR2 or CD14: *P<*0.001) and TGMs stimulated with 1 μg/ml of rBanLec (Fig 7F; with vs. without blocking TLR2 or CD14: *P<*0.001).

## Discussion

In this paper, we present that rBanLec binds murine peritoneal macrophages and modulates their functional characteristics. To the best of our knowledge, this is the first study addressing the specific influence of any BanLec isoform on macrophages’ functional characteristics depending on their previous experience, and showing BanLec interaction with TLR2 and CD14.

Peritoneal macrophages can be enriched from peritoneal lavage fluid due to their high adhesive capacity. In that way macrophage population is significantly deprived from other cells population which reside peritoneal cavity (B cells, dendritic cells, eosinophils, mast cells, neutrophils, T cells, NK cells, invariant NK T cells). Under resting conditions LPMs account for the majority of peritoneal macrophages [8], while upon exposure to proinflammatory stimuli (e.g. thioglycollate, zymosan, bacterial infection) SPMs are dominant subset of macrophages within peritoneal cavity. Hence, studies on RMs mainly reflect behavior of LPMs while studies on TGMs reflect behavior of SPMs under specific conditions.

Production of ROS and RNS are hallmark of macrophages activity. ROS and RNS may exert direct microbicidal and tumoricidal effects, but also, through mutual interactions and interactions with various intra- and extra-cellular signaling molecules, they regulate immune response and physiological processes [29, 30]. Although being essential in protection against various types of infection and for creation of a protective inflammatory milieu, the excessive, uncontrolled, production of ROS and RNS results in tissue damage and is associated with numerous pathological conditions [31]. Based on the enhanced activity of MPO, an enzyme involved in ROS and hypochlorous acid generation [32, 33], in both rBanLec-stimulated RMs and TGMs from BALB/c mice, it could be expected that rBanLec augments antimicrobial potential of macrophages. On the other hand, the lower capacity of macrophages to reduce NBT (proportional to the availability of O_2_^-^) upon rBanLec stimulation implies on the initiation of self-limiting mechanisms for ROS production in both RMs and TGMs. Decreased availability of O_2_^-^ may result from the enhanced MPO activity [33], but it may also imply on eventual concomitant enhancement in superoxide-dismutase activity [34]. Besides, some portion of O_2_^-^ may be quenched with NO produced upon rBanLec stimulation [29].

It is generally accepted that the initiation of NO production requires the stimulation via innate receptors and inflammatory milieu i.e. IFNγ [9]. It is reported that RMs from BALB/c and C57BL/6 mice (i) require different specific (co)stimulation via innate receptors and IFNγ to enhance NOS activity i.e. NO production in RMs from BALB/c and C57 BL/6, and (ii) set up significantly different net arginine metabolism (NOS vs. arginase activity) in the response to the same stimuli.

Per our results, rBanLec, due to its binding characteristics, could trigger signaling via innate receptors, while IFNγ can be produced mainly by macrophages, upon (autocrine) action of IL-12 [35–37]. Our results imply that upon rBanLec stimulation, irrespectively to the macrophages origin and type, pro-inflammatory signal primarily drive NO production as the intensity of NO production positively correlated with the IL-12 concentration in all tested systems (macrophages of the same type and genetic background). Although in all rBanLec-stimulated systems arginase activity (produced urea) was regulated in an opposite dose-dependent way comparing to the NO production, the strain-specific differences between the ratios of NOS and arginase activity were marked. We hypothesize that rBanLec stimulation, in a positive dose-dependent manner, augments proinflammatory potential in RMs (enhancement of NO production paralleled by reduction of arginase activity) but, in line with already reported results [9], in RMs from BALB/c mice, arginase activity was dominant, while in RMs from C57BL/6 dominated NOS activity.

The impact of genetic background on the NO-related outcome of rBanLec-stimulation seems to be more pronounced with TGMs. rBanLec stimulation, in a positive dose-dependent manner, enhanced proinflammatory potential of TGMs from C57BL/6 mice, while, in the same manner, promoted anti-inflammatory characteristics in TGMs from BALB/c mice. In other words, rBanLec, in a positive dose dependent manner, promoted the acquirement of inherent ratio of NOS and arginase activities, which was compromised by thyoglicollate treatment.

The specific ratio of NOS and arginase activities could be one of the explanations for differences in the proliferative responses of RMs and TGMs upon rBanLec stimulation. The NO, depending on its concentration, could be cytotoxic while arginase activity comprises to the cell growth and division [38]. In all rBanLec stimulated RMs cultures from BALB/c mice, arginase activity dominated over the NOS activity and it was higher comparing to the corresponding non-stimulated cells. In 10 μg/ml rBanLec–stimulated RMs from BALB/c mice (the lowest PI among rBanLec-stimulated RMs), arginase activity was significantly lower than in RMs from BALB/c mice stimulated with 1 μg/ml of rBanLec. In addition, 10 μg/ml rBanLec–stimulated RMs from BALB/c mice produced significantly higher amount of NO than the RMs stimulated with ten times lower concentration of rBanLec. Further, more significant dose-dependent changes in the ratio of NOS and arginase activities as well as more drastic dose-dependent differences in proliferative responses upon rBanLec stimulation were recorded for TGMs from comparing to RMs from BALB/c mice; the most proliferation-favorable ratio of NOS and arginase activities was recorded for TGMs stimulated with 10 μg/ml rBanLec, in the same cell culture where the highest proliferation index was recorded. The results related to the proliferation of RMs from C57BL/6 mice (S1 Fig) are in line with the explanation of the rBanLec-induced proliferation by the ratio of NOS and arginase activities). However, results obtained for rBanLec-stimulated TGMs from C57BL/6 mice, where both proliferation indices and domination of NOS activity positively correlated to the rBanLec concentration, imply that characteristics of arginine metabolism could not be the sole explanation for proliferation of macrophages upon rBanLec stimulation.

The binding characteristics of rBanLec (multiband reactivity on immunoblot, TLR2 and CD14 are spotted as some of target structures) and cytokine milieu recorded upon *in vitro* stimulation of peritoneal RMs and TGMs with rBanLec imply on the dominant occurrence of type II-like activation of macrophages [4]. Secretion of TNFα paralleled by dose-dependent enhancement of IL-10 secretion, characteristic for type II activated macrophages, has been marked with all rBanLec stimulated macrophages irrespective to their genetic background. However, cytokine profiles secreted by RMs and TGMs with the same genetic background were different, which confirms macrophages’ plasticity [1,5].

The comparison of effector and regulatory cytokines between macrophages of the same type but with different genetic background revealed that there is no cytokine profile which could be exclusively associated with rBanLec-stimulated either RMs or TGMs. This finding emphasizes the importance of genetic background when studying macrophage functional characteristics upon stimulation. For example, based only on the results obtained from BALB/c macrophages, we could conclude that rBanLec differently influence IL-12 (pro-Th1) and IL-4 (stimulator and marker of Th2 type of immune response) secretion, which could consequently cause different Th skewing when rBanLec-stimulated RMs and TGMs participate in shaping of the adaptive immune response. However, the same conclusion cannot be applied to C57BL/6-originated macrophages as rBanLec, in a positive dose-dependent manner, enhanced the production of both IL-4 and IL-12 by RMs and TGMs from C57BL/6 mice.

Besides, cytokine profiles recorded in analyzed cultures imply that various regulatory mechanisms are included to prevent exaggerated inflammatory response upon rBanLec stimulation and their different contribution to the balancing of inflammatory response under specific (activation state and origin of macrophages) conditions. First, the regulation based on the mutual influences of effector cytokines could be expected upon rBanLec stimulation. It is well-known that Th2 cytokines (IL-4) in certain situations inhibit the production of Th1 cytokines (IL-12) and *vice versa*, but the (autocrine) action of IL-4 is not always necessary or might be insufficient for the inhibition of IL-12 production. The decrease in IL-12 production might happen due to the anti-inflammatory action of TNFα [39]. Namely, it is shown that macrophages obtained under inflammatory conditions (such as TGMs), are susceptible to TNFα anti-inflammatory activity, which is accomplished through the inhibition of transcription of IL-12p40 subunit. Second, the productions of regulatory cytokines were specifically modulated by rBanLec stimulation. IL-10 and TGFβ are cytokines, which can exert anti-inflammatory mode of action [40]. TGFβ and IL-10 when acting synergistically exert more pronounced anti-inflammatory effects than each of them, in equivalent amount, promote separately [41]. Also, already mentioned TNFα inhibitory influence on IL-12 production by TGMs could be enhanced by TGFβ [42]. Finally, anti-inflammatory influences of TGFβ and IL-10 as well as IL-4 based on the inhibition of NO production and concomitant enhancement of arginase activity are reported as well [40, 43].

If we take the rBanLec dose-dependent pattern of changes in NO production within a specific system (macrophages of the same origin and type) as an indicator for the prevalent activity of proinflammatory cytokines, three main conclusions could be drawn. First, RMs from BALB/c mice upon rBanLec stimulation in a positive dose-dependent manner augment proinflammatory capacity primarily due to the enhancement of IL-12 production and concomitant reduction in production of IL-4 and TGFβ, while positive dose-dependent enhancement in IL-10 tend to prevent exaggerated inflammatory response. Second, lessening the proinflammatory capacity of TGMs from BALB/c mice by increasing stimulatory rBanLec dose results from the decrease in IL-12 production and concomitant enhancement of IL-4, TGFβ and IL-10 production. Third, despite positive dose-dependent enhancement of IL-4, TGFβ and IL-10 production, the domination of the effects of proinflammatory cytokines was more apparent in cultures of RMs and TGMs from C57BL/6 mice when the rBanLec stimulatory dose was increased.

Abovementioned cytokine pattern, reported analyses of TLR2, TLR4 and its co-receptor CD14 protein sequences (implying on several amino-acid residues as potential spots for N-glycosylation [14]) and confirmed occurrence of N-linked mannose-rich oligosaccharides on mouse TLR2 [17, 44–46], TLR4 [17, 45] and CD14 [46, 47] lead us to envisage that rBanLec might bind these structures. Our observation that rBanLec does not bind TLR4 but binds TLR2 and CD14 by forming interactions which can be prevented by addition of mannose is in line with the results reported for ArtinM, a mannose-specific lectin isolated from *Artocarpus heterophyllus* (jackfruit). Mariano et al. showed that ArtinM binds TLR2 in a carbohydrate recognition-dependant manner and that this is crucial for its immunomodulatory activity, including the enhancement in production of IL-10 and IL-12p40 by RMs [14]. We have further shown that interactions of rBanLec with TLR2 and CD14 are important for initiation of TNFα secretion and NOS activity. The blockades of CD14 exerted significant negative impact on IL-10 production only with TGMs, which may be explained by a variety of signals that could promote IL-10 production in macrophages [48] and their specific contribution to overall IL-10 promotion depending on the functional state of the target cell. Finally, our results are in line with numerous data showing that TLR ligation is important to produce mediators of inflammation [49].

In conclusion, we have unequivocally demonstrated that the outcome of rBanLec stimulation depends on rBanLec concentration as well as on the functional characteristics of its target cells and their genetic background. Before considering rBanLec as an eventual therapeutic, it would be of utter importance not only to investigate its effects under physiological conditions but to include rBanLec immunomodulatory activity within specific pathological conditions.

## Supporting information

S1 Fig Proliferation index (PI) of rBanLec-stimulated peritoneal RMs and TGMs from C57BL/6 mice. Macrophages were incubated either without any stimulation or with 1, 5 and 10 μg/ml rBanLec. Number of viable cells was determined by CCK-8 assay after 48h long incubation and used for PI calculations. The results were presented as mean PI ± SE. The significance of the observed differences was calculated by one-way repeated ANOVA followed by Bonferroni’s multiple comparison test (*P* <0.05*, *P* <0.005**, *P* <0.0001***). Solid lines indicate compared groups.
(TIF)

S2 Fig Control staining for TLR2, TLR4, CD14 and rBanLec as well as appropriate isotype controls. TLR2, TLR4 and CD14 were detected with specific FITC-labeled monoclonal antibodies (green color) and rBanLec (biotin-labeled) was detected with streptavidin-rhodamineB (red color). Isotype-matched controls for each antibody as well as streptavidin-rhodamine were used for negative staining controls.
(TIF)

S3 Fig Western blot analysis of rBanLec binding to proteins in TGMs lysate. Whole cell lysate was prepared after collecting TGMs from peritoneum, resolved on 9% polyacrylamide gel by non-reducing SDS-polyacrylamide gel electrophoresis and transferred onto PVDF membrane. Binding of biotin-labeled rBanlec to the proteins from TGMs lysate was visualized with extrAvidine-alkaline phosphatase / 5-Bromo-4-chloro-3-indolyl phosphate/NBT system.
(TIF)

S4 Fig **Western blot detection of TLR2 (A), TLR4 (B) and CD14 (C) in cell lysate prepared from peritoneal TGMs.** Whole cell lysate was prepared after collecting TGMs from peritoneum, resolved on 9% polyacrylamide gel by non-reducing SDS-polyacrylamide gel electrophoresis and transferred onto PVDF membrane. TLR2, TLR4 and CD14 were detected using the specific biotin-labeled monoclonal antibodies. extrAvidine-alkaline phosphatase /5-Bromo-4-chloro-3-indolyl phosphate/nitro-blue tetrazolium chloride system was used for visualization.
(TIF)
