# Supplementary figures and images for: Modulation of functional characteristics of resident and thioglycollate-elicited peritoneal murine macrophages by a recombinant banana lectin
Source: PLoS One. 2017 Feb 24;12(2):e0172469. doi: 10.1371/journal.pone.0172469 (PMC5325268; doi:10.1371/journal.pone.0172469)

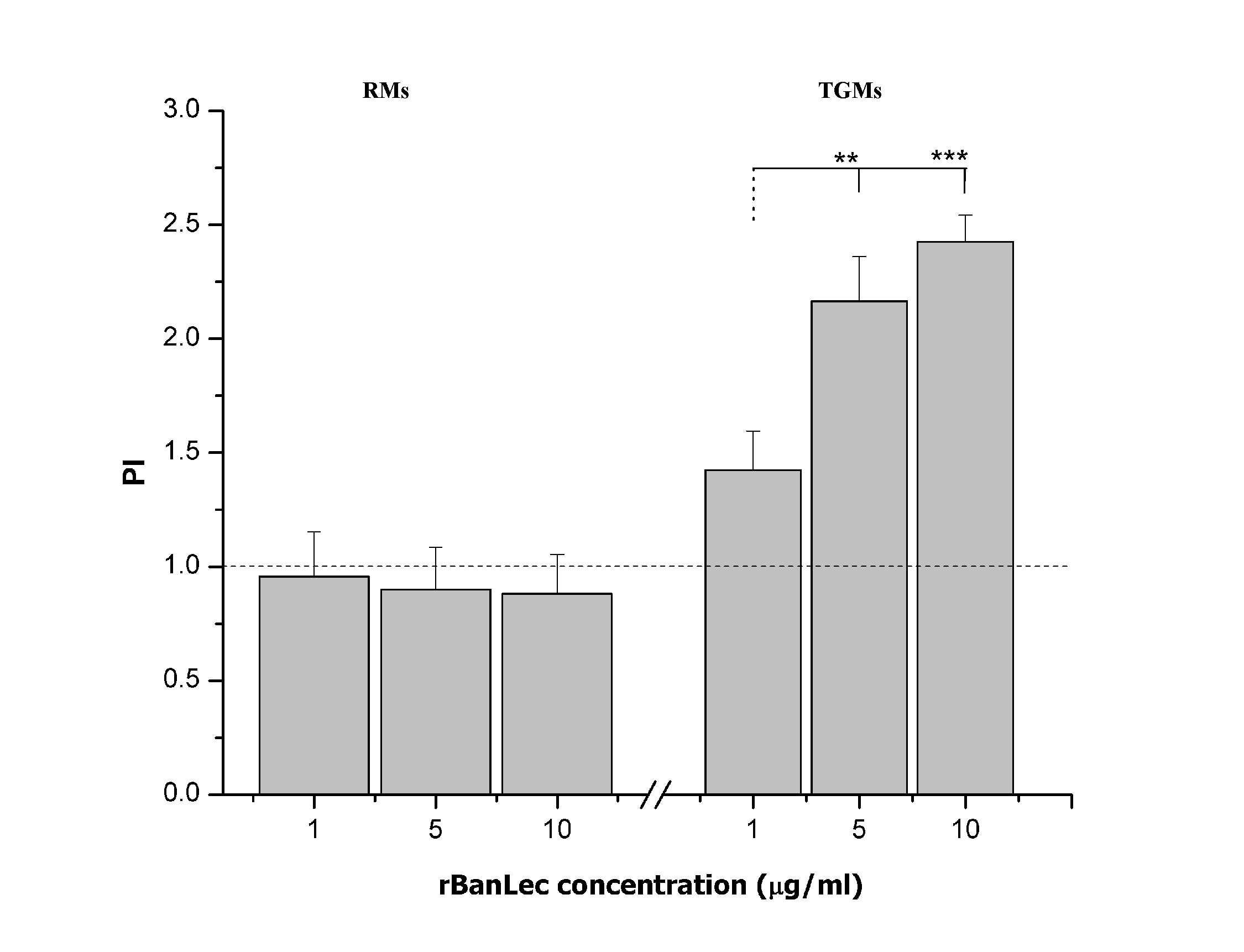

Supplement: S1 Fig — Macrophages were incubated either without any stimulation or with 1, 5 and 10 μg/ml rBanLec. Number of viable cells was determined by CCK-8 assay after 48h long incubation and used for PI calculations. The results were presented as mean PI ± SE. The significance of the observed differences was calculated by one-way repeated ANOVA followed by Bonferroni’s multiple comparison test (P <0.05*, P <0.005**, P <0.0001***). Solid lines indicate compared groups. (TIF) [file pone.0172469.s001.tif]

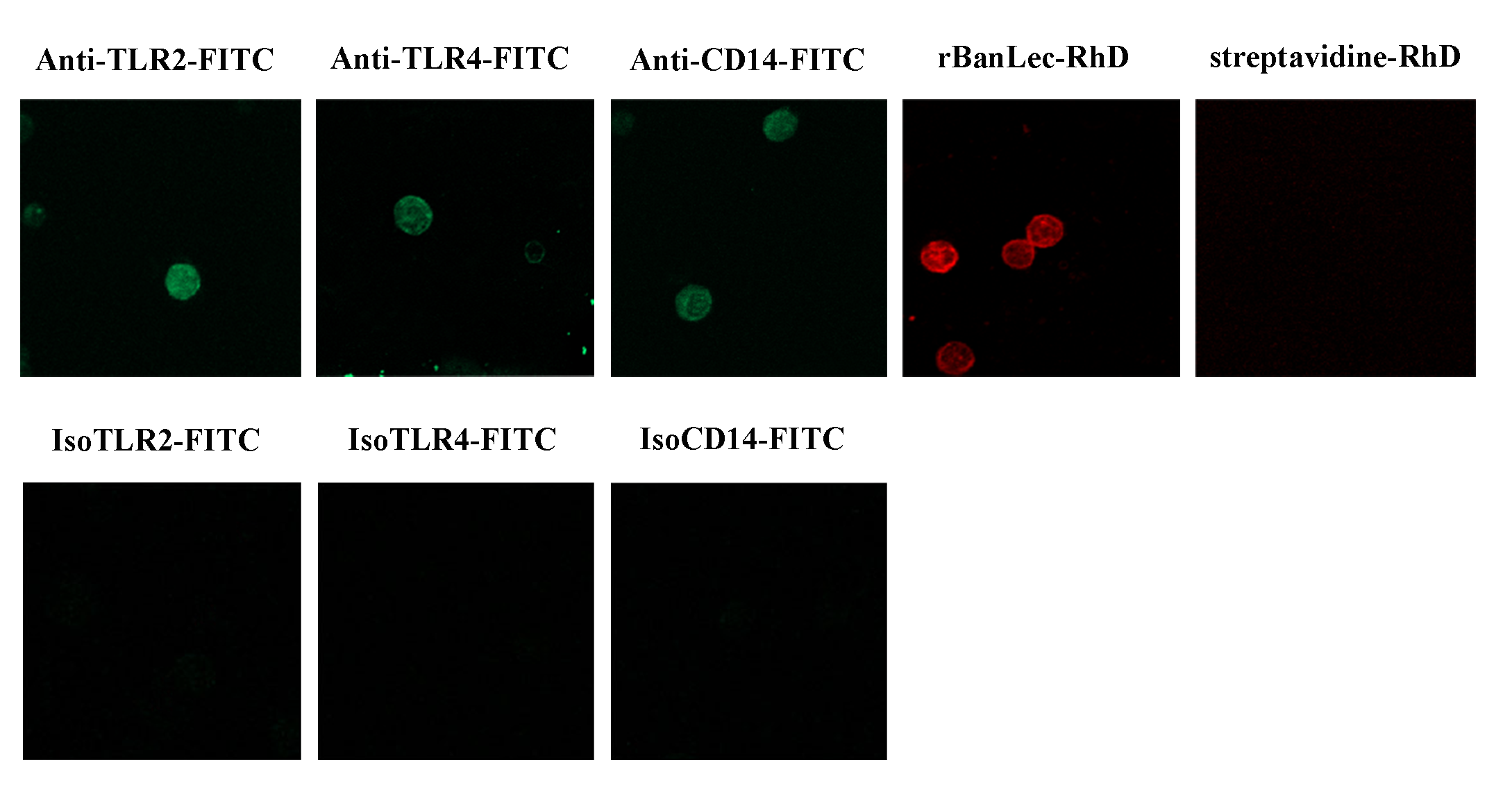

Supplement: S2 Fig — TLR2, TLR4 and CD14 were detected with specific FITC-labeled monoclonal antibodies (green color) and rBanLec (biotin-labeled) was detected with streptavidin-rhodamineB (red color). Isotype-matched controls for each antibody as well as streptavidin-rhodamine were used for negative staining controls. (TIF) [file pone.0172469.s002.tif]

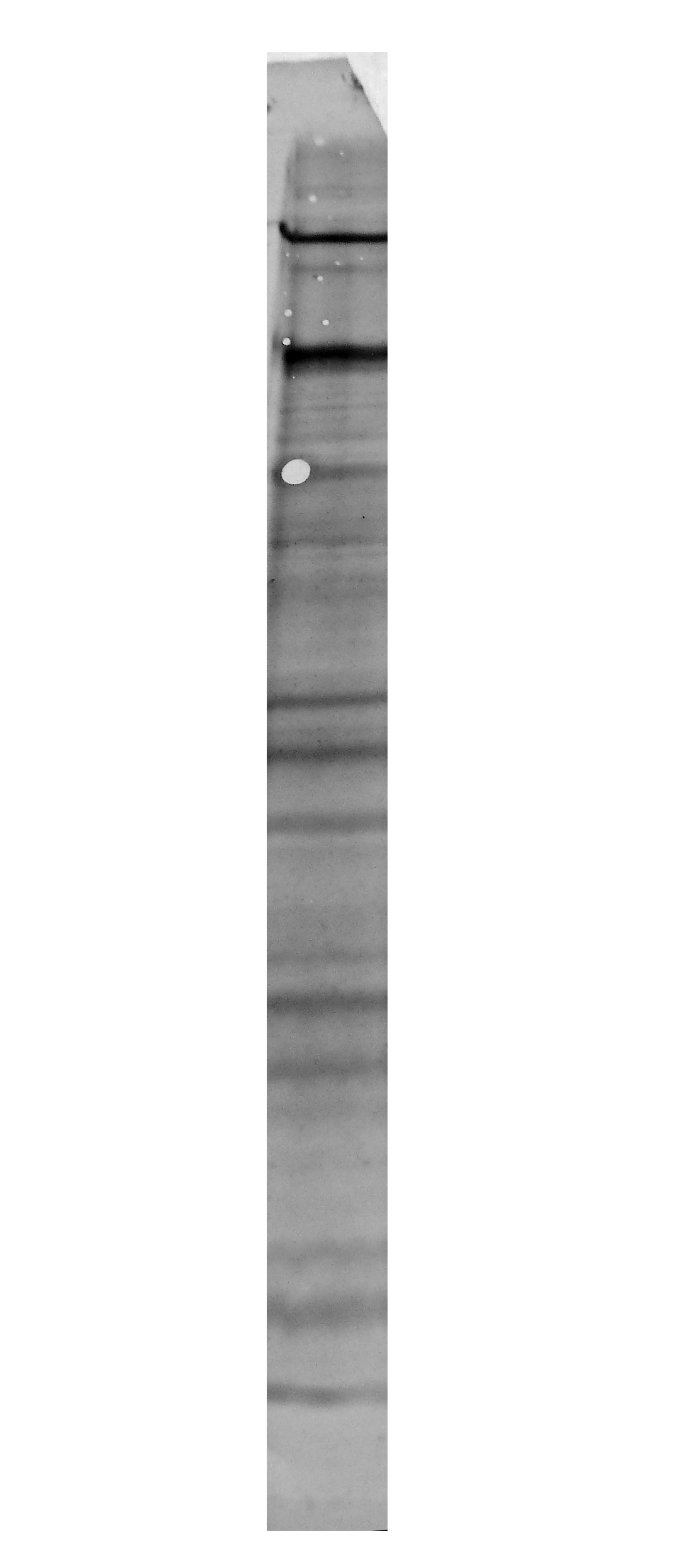

Supplement: S3 Fig — Whole cell lysate was prepared after collecting TGMs from peritoneum, resolved on 9% polyacrylamide gel by non-reducing SDS-polyacrylamide gel electrophoresis and transferred onto PVDF membrane. Binding of biotin-labeled rBanlec to the proteins from TGMs lysate was visualized with extrAvidine-alkaline phosphatase / 5-Bromo-4-chloro-3-indolyl phosphate/NBT system. (TIF) [file pone.0172469.s003.tif]

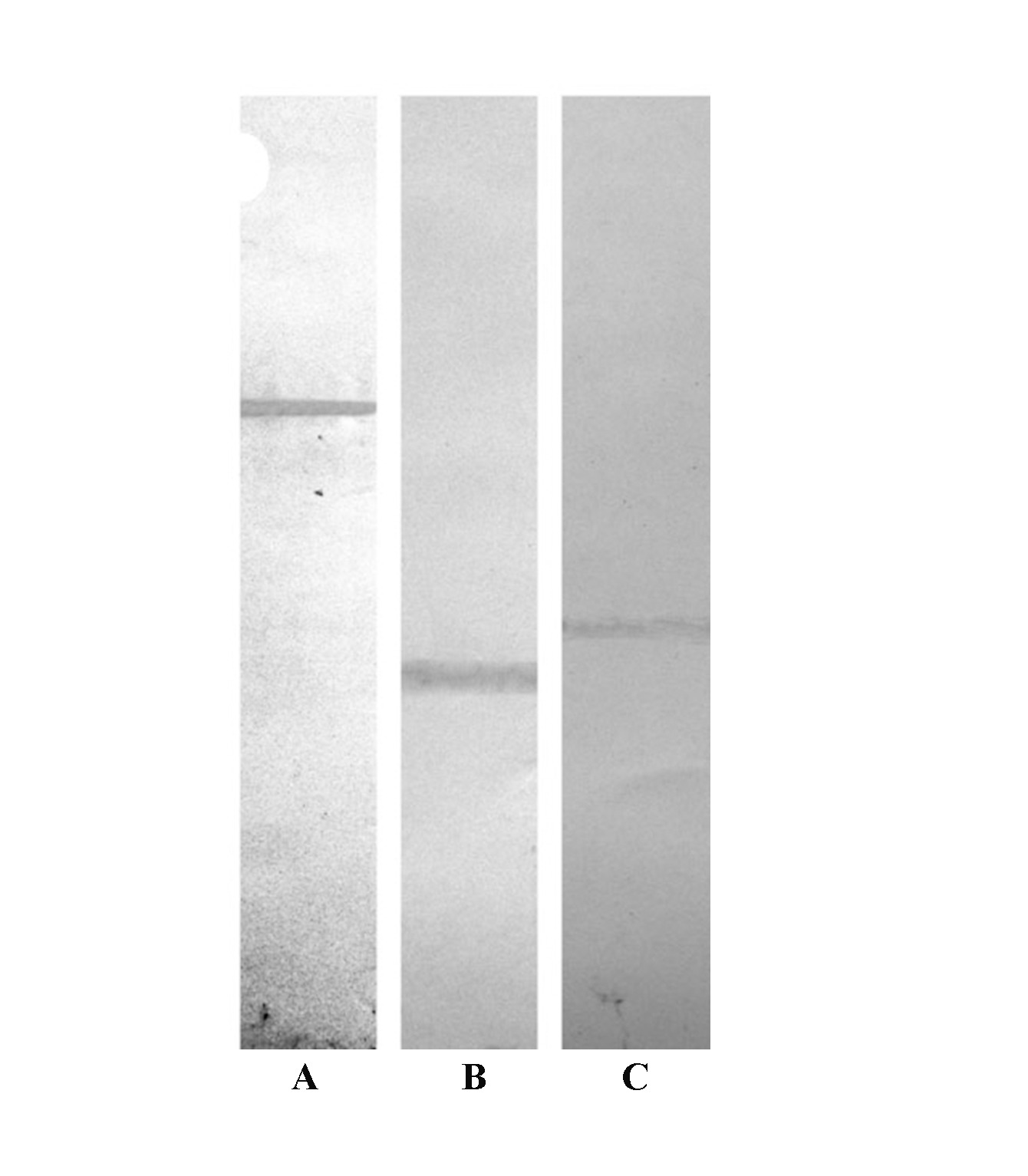

Supplement: S4 Fig — Western blot detection of TLR2 (A), TLR4 (B) and CD14 (C) in cell lysate prepared from peritoneal TGMs. Whole cell lysate was prepared after collecting TGMs from peritoneum, resolved on 9% polyacrylamide gel by non-reducing SDS-polyacrylamide gel electrophoresis and transferred onto PVDF membrane. TLR2, TLR4 and CD14 were detected using the specific biotin-labeled monoclonal antibodies. extrAvidine-alkaline phosphatase /5-Bromo-4-chloro-3-indolyl phosphate/nitro-blue tetrazolium chloride system was used for visualization. (TIF) [file pone.0172469.s004.tif]
